# Does the Digital Therapeutic Alliance Exist? Integrative Review

**DOI:** 10.2196/69294

**Published:** 2025-02-07

**Authors:** Amylie Malouin-Lachance, Julien Capolupo, Chloé Laplante, Alexandre Hudon

**Affiliations:** 1 Département de psychiatrie, Université Laval, Québec City, QC, Canada; 2 Faculté de Médecine, Université Laval, Québec City, QC, Canada; 3 Department of Psychiatry, Institut universitaire en santé mentale de Montréal, Montreal, QC, Canada; 4 Centre de recherche de l'Institut universitaire en santé mentale de Montréal, Montreal, QC, Canada; 5 Department of Psychiatry, Institut nationale de psychiatrie légale Philippe-Pinel, Montreal, QC, Canada; 6 Department of Psychiatry, Faculty of Medicine, University of Montreal, Montreal, QC, Canada

**Keywords:** psychotherapy, mental health, psychiatry, artificial intelligence, therapeutic alliance, digital interventions, chatbot, psychology

## Abstract

**Background:**

Mental health disorders significantly impact global populations, prompting the rise of digital mental health interventions, such as artificial intelligence (AI)-powered chatbots, to address gaps in access to care. This review explores the potential for a “digital therapeutic alliance (DTA),” emphasizing empathy, engagement, and alignment with traditional therapeutic principles to enhance user outcomes.

**Objective:**

The primary objective of this review was to identify key concepts underlying the DTA in AI-driven psychotherapeutic interventions for mental health. The secondary objective was to propose an initial definition of the DTA based on these identified concepts.

**Methods:**

The PRISMA (Preferred Reporting Items for Systematic Reviews and Meta-Analyses) for scoping reviews and Tavares de Souza’s integrative review methodology were followed, encompassing systematic literature searches in Medline, Web of Science, PsycNet, and Google Scholar. Data from eligible studies were extracted and analyzed using Horvath et al’s conceptual framework on a therapeutic alliance, focusing on goal alignment, task agreement, and the therapeutic bond, with quality assessed using the Newcastle-Ottawa Scale and Cochrane Risk of Bias Tool.

**Results:**

A total of 28 studies were identified from an initial pool of 1294 articles after excluding duplicates and ineligible studies. These studies informed the development of a conceptual framework for a DTA, encompassing key elements such as goal alignment, task agreement, therapeutic bond, user engagement, and the facilitators and barriers affecting therapeutic outcomes. The interventions primarily focused on AI-powered chatbots, digital psychotherapy, and other digital tools.

**Conclusions:**

The findings of this integrative review provide a foundational framework for the concept of a DTA and report its potential to replicate key therapeutic mechanisms such as empathy, trust, and collaboration in AI-driven psychotherapeutic tools. While the DTA shows promise in enhancing accessibility and engagement in mental health care, further research and innovation are needed to address challenges such as personalization, ethical concerns, and long-term impact.

## Introduction

Mental health disorders represent a significant public health burden, affecting around 13% of the global population annually [1]. These conditions lead to an important human and economic impact, including a diminished quality of life, decreased productivity, and increased health care costs [2,3]. As an example, in Canada, this increase in demand is combined with multiple barriers to accessing mental health services, such as costs, lack of information, long waiting times, and insufficient funding, which all lead to reduced access to care [4]. This affects vulnerable populations disproportionately, leaving many without timely or adequate treatment [5]. As the demand for mental health services continues to exceed the ability of care, innovative approaches are needed to address this gap.

In the last years, digital mental health interventions (DMHIs), particularly mental health applications and chatbots, have emerged to complement conventional therapeutic models [6]. Many of them leverage artificial intelligence (AI) paired with evidence-based psychotherapeutic frameworks, such as cognitive-behavioral therapy (CBT) or mindfulness-based interventions [7]. Engagement and adherence to digital treatment was generally considered low in past studies, but the integration of factors such as human support and personalization led to better outcomes [8-11]. Chatbots gained attention for their ability to deliver support in a flexible and user-friendly format with a conversational approach mimicking human interaction. They showed effectiveness in improving depression and anxiety in multiple reviews and meta-analyses with moderate to large effect sizes, but limitations were noted, such as a high risk of bias and lack of long-term follow-up [12-14].

When designing chatbots to effectively address mental health problems through a psychotherapeutic approach, it seems essential to understand some of the mechanisms underlying the effectiveness of psychotherapy. Many authors explored the role of the therapeutic alliance in this matter, and regardless of the approach, it emerges as a cornerstone of effective treatment [15-17]. Zetzel, an important figure in the field of psychoanalysis, believed the therapeutic alliance reflected the collaborative state between a patient and the therapist that facilitates treatment [18,19]. Authors such as Hausner [18] pushed this comprehension by distinguishing between the therapeutic alliance and the working alliance. The therapeutic alliance seemed to be made by “mutual identification, empathy and role-responsiveness,” while the working alliance could only be possible “after a therapeutic alliance has to some degree been established” [18].

Considering the clear role of these concepts in psychotherapy efficacy, this raises important questions concerning the application of chatbots in mental health. With a design made to simulate human interaction, is it possible to nurture a meaningful alliance between the chatbot and the user? While most of the studies focus on satisfaction and engagement metrics, few of them analyze all the components of a therapeutic alliance [12,20,21]. Empathy appears to be a recurring interest in studies and an important factor for the development of an alliance with a chatbot by creating a sense of warmth [22]. On that topic, Boucher et al [23] pointed out that “chatbots designed to display empathetic reactions are rated more positively (ie, more enjoyable, understanding, sociable, trustworthy, and intelligent) than one that is not programmed to respond empathetically.” With the rise of generative AI, which refers to complex mathematical algorithms that can learn from large data sets to produce text, audio, or visuals that resemble those of a human, this is even more pertinent [24]. Considering these, algorithms are currently studied as to their potential integration in mental health treatments to create AI-powered chatbots and digital therapists that offer people quick, individualized support, personalized interactions between the machines and humans must be further defined [24].

These preliminary findings, especially on empathy, hint at the possibility of a “digital therapeutic alliance (DTA),” which some have tried to grasp its scope using existing scales such as the Working Alliance Inventory-Short Revised (WAI-SR) [25]. To our knowledge, there is no standardized concept that defines and evaluates the presence of an alliance between a chatbot and its user, and if the same components as with the human therapist apply. Understanding how this bond can emerge might be a key factor in boosting engagement and designing more impactful DMHIs. The main objective of this review was to identify the key concepts in a DTA between AI-driven psychotherapeutic interventions and the user in the context of mental health interventions. The secondary objective is to provide an initial definition of DTA using these concepts. It is hypothesized that components of a DTA will align with those found in traditional human-therapist alliances but will manifest differently due to the nonhuman nature of AI-driven psychotherapeutic interventions. Furthermore, it is possible that higher levels of perceived empathy and trust within AI-driven psychotherapeutic interventions will predict greater user engagement and adherence to DMHIs, similar to the dynamics observed in human-therapist relationships.

## Methods

### Search Strategies

The PRISMA (Preferred Reporting Items for Systematic Reviews and Meta-Analyses) for scoping reviews methodology was conducted to provide a comprehensive overview of the key concepts in DTA between AI-driven psychotherapeutic interventions and the user in the context of mental health interventions. The methodology also followed the methodology developed by Souza and Silva [26] for integrative reviews. This approach includes 6 steps: the development of the research question, literature search, data collection, critical analysis of the identified articles, discussion of the results, and presentation of the integrative perspective of the identified articles [26].

The literature review was conducted in collaboration with a librarian specialized in mental health. The databases Medline, Web of Science, PsycNet (PsycINFO), and Google Scholar were searched to retrieve articles published from their inception up to December 2024. These databases were selected with the help of an experienced librarian in the field of mental health to ensure comprehensive coverage of biomedical, psychological, and multidisciplinary literature relevant to digital interventions and generative AI in mental health care. Keywords and indexing terms related to AI, therapeutic alliance, and digital interventions were used. The literature search was carried out by all the authors (AML, JC, CL, and AH). Complete search strategies are available in Multimedia Appendix 1 and the PRISMA for Scoping Reviews checklist is also provided as Multimedia Appendix 2.

### Study Eligibility

The reviewed studies were included in the analysis if they met the following inclusion criteria: (1) the main topic of interest was on a psychotherapeutic intervention; (2) the study was conducted in the field of psychiatry or mental health; (3) the psychotherapeutic intervention used AI as part of its design or included a data-driven approach; and (4) the manuscript was written in French or English. Case studies, protocols, pre-experimental studies, and unpublished writings were excluded from the analysis.

### Data Extraction

Data were extracted using a standardized Microsoft Excel form. The studies identified were independently counter-verified for consistency and integrity by 2 authors (AML and AH). Any disagreements regarding the inclusion or exclusion of a study were mutually resolved by the authors. The extracted information included authors, population (sample), type of interventions, type of engagement, facilitators, challenges, and outcomes.

### Data Analysis

All the studies were analyzed according to the conceptual framework developed by Horvath and Luborsky [15] about the key components usually found in a therapeutic alliance. This framework, designed to understand further the alliance between a therapist and a patient, defines three key components: (1) agreement on therapeutic goals, (2) agreement on therapeutic tasks, and (3) the therapeutic bond.

A key component of successful treatment is the client and therapist’s agreement on therapeutic goals, which promotes cooperation, trust, and mutual understanding [27]. Achieving therapeutic success depends on both parties working toward meaningful and mutually acknowledged goals, which is ensured by this alignment. Clients are more inclined to participate actively in the process, which increases motivation and commitment, when they believe their goals are recognized and understood [28]. Additionally, goal agreement reduces misconceptions, creates a sense of direction, and clarifies expectations [28]. Because it strengthens the therapeutic bond, which is essential to effective interventions, research continuously shows that agreement on therapeutic goals is a strong predictor of beneficial outcomes across a variety of therapeutic modalities [29].

Another important component of the therapeutic process is the agreement on therapeutic tasks, which implies that the client and the therapist agree on the methods, exercises, and interventions needed to meet the goals of the therapy [30]. This mutual comprehension improves teamwork and gives patients a sense of empowerment and involvement in their healing process. Task agreement also enables therapists to customize interventions to the client’s preferences and needs, enhancing the therapy’s efficacy and relevance [31]. Additionally, studies show that the therapeutic alliance, which is closely linked to successful outcomes, depends on alignment on therapeutic activities [32].

Finally, the primary element of the therapeutic relationship is the therapeutic bond, which includes the client and therapist’s mutual regard, trust, and emotional connection [33]. A secure and encouraging atmosphere where clients feel appreciated, understood, and free to express their feelings is built on this connection. Strong therapeutic relationships promote cooperation, increase the client’s sense of trust in the therapist, and promote candor and openness [33]. Because it provides clients with the comfort of a trustworthy and understanding ally, research continuously shows that the therapeutic relationship is a significant predictor of successful outcomes [34].

### Quality Assessment

The quality of the studies included in this analysis was assessed using 2 widely recognized tools: the Newcastle-Ottawa Scale for nonrandomized controlled studies and the Cochrane Risk of Bias Tool for randomized controlled trials [35,36]. The Newcastle-Ottawa Scale evaluates the quality of cohort and case-control studies by examining 3 key domains: selection of study groups, comparability between groups, and the ascertainment of either exposure or outcome. Each domain is associated with specific criteria, and studies earn stars for meeting these standards, with a maximum score of 9 stars representing the highest quality [35].

For randomized controlled trials, the Cochrane Risk of Bias Tool was employed to systematically assess potential biases. This tool examines 7 domains: random sequence generation, allocation concealment, participant and personnel blinding, outcome assessment blinding, completeness of outcome data, selective reporting, and other potential sources of bias [36]. Each domain is categorized as having a low, high, or unclear risk of bias based on predefined guidelines.

In this review, studies were categorized based on their quality as follows: studies receiving 1‐3 stars on the Newcastle-Ottawa Scale or rated as having a high risk of bias using the Cochrane tool were deemed low quality; those with 4‐6 stars or a moderate risk of bias were considered moderate quality; and those earning 7‐9 stars on the Newcastle-Ottawa Scale or demonstrating a low risk of bias were classified as high quality.

## Results

### Description of Studies

**Figure 1.**
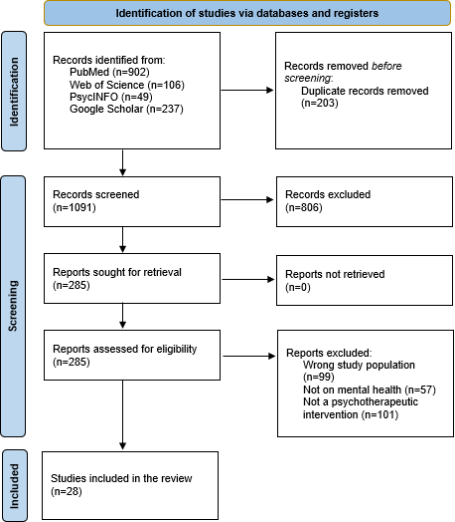
PRISMA (Preferred Reporting Items for Systematic Reviews and Meta-Analyses) flowchart for the inclusion of studies.

The literature review initially identified 1294 articles. Of these, 203 duplicates were removed. Among the remaining 1091 studies, a total of 806 articles were excluded after reviewing their titles and abstracts as they did not meet the inclusion criteria (307 were not targeting the specific population and 499 did not involve a psychotherapeutic intervention). A total of 28 articles were fully retained following the comprehensive analysis of the 285 articles selected for eligibility evaluation. Details of the article selection process are presented in Figure 1, and the identified articles are listed in Multimedia Appendix 3.

**Figure 2.**
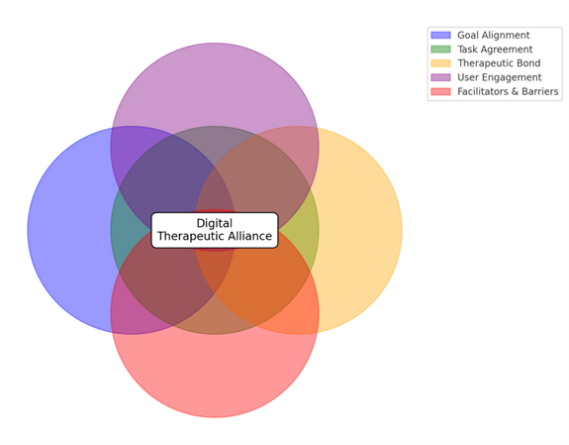
Key concepts to define the digital therapeutic alliance.

A conceptual framework to define the concept of DTA was developed by identifying and integrating the findings of the analyzed studies. The key concepts are all inter-connected and imply the following elements: goal alignment, task agreement, therapeutic bond, user engagement, as well as barriers and facilitators. Key concepts to define the DTA are presented in Figure 2.

From the 28 identified studies, 6 major themes regarding the type of interventions were found: AI-powered chatbots (n=12), digital psychotherapy (n=7), exploratory or review studies (n=3), general AI health care tools (n=2), personalized therapy (n=2), and behavioral change tools (n=2).

### Goal Alignment

A total of 16 studies reported the use of goal alignment in their data-driven intervention. Goal alignment refers to the idea that there must be an agreement over common goals or outcomes when initiating psychotherapy. While heterogenous, several approaches were identified with regard to goal alignment. Aggarwal et al [37] emphasized the integration of behavior change theories, including goal-setting frameworks, to improve both primary and secondary outcomes. Similarly, Beatty et al [25], He et al [38], and Jeong et al [39] highlighted the importance of measuring goal alignment using the WAI-SR subscale, which evaluates how users and the chatbot collaborate to set mental health-related goals. Entenberg et al [40] as well as Martinengo et al [41] reported in the case of their study that therapeutic goals were established by the chatbot at the beginning of the interaction, ensuring clarity from the outset. Forman-Hoffman et al [42] reported that in their context, therapeutic goal alignment was evaluated via the therapeutic alliance score, highlighting a structured measurement approach.

Like human-based therapeutic alliances, goal-setting mechanisms are important to provide effective therapeutic alliances within both digital and human contexts. In the absence of goal alignment between the digital intervention itself and the user, a few studies reported that this was done before the intervention was used with a human agent [43,44].

Another study reports that the alignment on therapeutic goals is mediated by emotion regulation self-efficacy, with digital and therapeutic alliances predicting symptom reduction through increased emotion regulation self-efficacy [45]. This is similar to Cross et al’s [46] study, which reported that new eHealth-specific therapeutic alliance subscales, such as perceived emotional investment and sense of relatedness, align users’ goals with therapy outcomes more effectively than conventional measures in digital parent training programs. In Doukani et al’s [47] work, alignment on therapeutic goals was higher in blended CBT compared to treatment-as-usual, with good system usability enhancing working alliance and leading to improved depression outcomes. Finally, in Brotherdale et al, it is demonstrated that the alignment of goals and expectations in fully automated digital mental health apps often involves lower expectations compared to in-person therapy but can be enhanced by personalized interfaces and validating features.

### Task Agreement

Agreement on therapeutic tasks refers to the mutual understanding and collaboration between the therapist (or digital tool) and the user on the specific activities or steps required to achieve therapeutic goals. A total of 8 studies provided insight on this key concept. Similarly to the findings identified in the goal alignment section, various approaches have been conducted to achieve task agreement among the analyzed studies. Beatty et al [25] evaluated therapeutic tasks using the WAI-SR subscale for tasks. Forman-Hoffman et al [42] assessed task agreement via the therapeutic alliance score. Goldberg et al [48] described task alignment as conducted between humans like in traditional therapy. Hocking et al [43] emphasized the use of the *Specific, Measurable, Achievable, Relevant, and Time-Bound* (SMART) goals framework for aligning tasks with therapeutic objectives. Martinengo et al [41] focused on task agreement as observed in human interactions, while Liu et al [44] explored task alignment through conversational agents. Similarly, Goldberg et al [49] reported that the agreement on therapeutic tasks and goals in unguided smartphone apps can be assessed using the Digital Working Alliance Inventory and showed positive predictions for app engagement and reductions in psychological distress. Collectively, these studies reported the importance of task agreement to enhance therapeutic alliances across both digital and human-mediated interventions.

### Therapeutic Bond

Therapeutic bond refers to the emotional connection and trust developed between the user and the therapist or digital tool. In total, 12 studies reported how they assessed the therapeutic bond between the human and the machine during digital interventions.

Beatty et al [25] reported that users expressed strong emotional connections in the first assessment, comparable to traditional in-person CBT, as measured by the WAI-SR subscale for the bond. Forman-Hoffman et al [42], as for the previous components, evaluated the bond using the therapeutic alliance score. He et al [38] and Jeong et al [39] measured the bond using the WAI-SR.

Interestingly, Liu et al [44] found that conversational AI showed higher therapeutic alliance scores compared to bibliotherapy with a Cohen *d*=0.83. MacNeill et al [20] noted that while some users felt reassured and connected, others experienced difficulty establishing a bond due to conversational issues. Liu et al [44] observed that an empathic tone and conversational nature facilitated engagement but that rule-based systems limited the bond. Plakun [50] highlighted that AI struggles to replicate the emotional depth and transference found in human relationships, limiting its ability to build authentic therapeutic bonds. Russo et al [51] reported that therapists found the therapeutic bond with AI tools the most challenging aspect of therapy, as it is difficult to establish. Interestingly, in Prescott and Hanley’s [52] review, it was observed that elders were more expressive with sociable robots than with task-oriented ones when comparing groups. Finally, Ta-Johnson et al [53] described how users build confidence with chatbots, using them as a safe space to discuss feelings and develop a bond resembling a therapeutic relationship.

### User Engagement

The concept of user engagement refers to the level of sustained interaction, interest, and participation between the user and the therapeutic tool. Insights into this key concept were reported in 22 studies, highlighting a range of factors influencing engagement.

Aggarwal et al [37] found that engagement was primarily established through the frequency of messages exchanged between the chatbot and the user, though this varied across studies. Alfano et al [54] emphasized that initial engagement was high, driven by ease of access and the perception of nonjudgmental interactions, but noted that dropout rates increased when users did not experience quick results. Anisha et al [55] observed that anthropomorphic conversational agents (CAs) were preferred, leading to higher intervention compliance compared to mechanical chatbots. Similarly, Beatty et al [25] reported that 73.8% of participants continued using the Wysa chatbot after the first assessment, with engagement improving over time and correlating with an increased bond subscore in later assessments.

Several studies explored how engagement was measured or facilitated. Entenberg et al [40] described engagement as determined by the number of messages and characters sent. Escobar Viera et al [56] found that participants sent an average of 49.3 messages to the chatbot, highlighting message frequency as an indicator of engagement. Forman-Hoffman et al [42] evaluated engagement through app utilization rates, which varied across conditions. Goonesekera and Donkin [57] noted that while daily check-ins were appreciated, some participants found them tedious due to competing social engagements, fatigue, and daily responsibilities.

Specific tools and features also played a role in user engagement. He et al [38] used the *User Engagement Scale* and found higher interaction levels with motivational-interviewing chatbots. Hocking et al [43] observed stable engagement among 2 patients using the *Rehabilitation Therapy Engagement Scale*. Inkster et al [58] measured engagement based on the number of active session days between screenings. Kettle and Lee [59] identified key drivers of engagement, including agent connection, initial motivation (eg, curiosity or loneliness), and technical features like accessibility. Liu et al [44] noted that rule-based conversational agents often provided inadequate responses, frustrating users and reducing engagement.

Furthermore, in eHealth interventions, novel engagement techniques, including perceived emotional investment and application-induced accountability, were particularly useful. As though speaking with a supportive spouse, these tactics urged users to remain dedicated and sense a connection to the digital instrument. Accessibility and empowerment were reported as important for standalone apps, enabling users to interact at their own comfort level and at their own pace [60]. Furthermore, the application of innovative metrics such as the *eHealth Therapeutic Alliance Inventory* and the *Digital Working Alliance Inventory* showed that engagement could be quantitatively associated with outcomes like improved behavior change, decreased psychological distress, and app usage [49,61].

Finally, studies highlighted broader implications of user engagement. Russo et al [51] emphasized that digital tools could improve access for hard-to-reach individuals, reducing inequalities in therapy. Ta-Johnson et al [53] described how users engaged steadily with chatbots across various topics, often returning to share updates about their emotions and experiences, reflecting the potential for long-term engagement.

### Facilitators and Barriers

A DTA can also be defined by its variety of facilitators and barriers that intersect every other mentioned key concept. In total, 22 studies reported either a facilitator, a barrier, or examples for both categories. Facilitators and barriers refer to the factors that enhance or hinder the effectiveness of digital therapeutic tools, ranging from technological features to user perceptions.

Aggarwal et al [37] identified facilitators such as free-flow conversations, which enhanced user experience through personalization, while Alfano et al [54] highlighted the role of comfort with digital technology, particularly among adolescents. Anisha et al [55] emphasized strong social presence and emotional closeness as key facilitators for positive outcomes but noted that scripted chatbots often failed to provide personalized or empathetic responses, creating user frustration. Furthermore, the ability to generate emotion regulation self-efficacy was an important facilitator. By enhancing users’ confidence in managing emotions, digital tools increased motivation to engage and persist in the therapeutic process [45,49]. Beatty et al [25] reported that anonymity and flexibility raised user autonomy but highlighted challenges with chatbots’ limited understanding of user needs, leading to perceived ineffectiveness.

Chan et al [62] discussed issues with inappropriate chatbot reinforcements, context misunderstandings, and technical errors, which undermined their reliability. Entenberg et al [40] noted that customizable and human-like features significantly enhanced user satisfaction, while Forman-Hoffman et al [42] highlighted design elements like responsiveness and inclusivity (eg, avoiding past names for trans users) as facilitators. However, limited conversational content and inability to handle complex interactions were key barriers [56]. Goonesekera and Donkin [57] identified anthropomorphic features and engaging content as key facilitators but pointed to technical difficulties and limited interactivity in decision-tree-based chatbots as barriers. Also, Brotherdale et al’s [60] and Ashur et al [61] studies reported the flexibility, choice, and empowerment that standalone apps provided allowed users to engage with the intervention at their own comfort level and at their own speed. Mobile app accessibility decreased stigma and offered anonymity, especially for populations that are difficult to reach.

Jeong et al [39] observed that users appreciated engaging wellness activities and positive reinforcement but struggled to adapt to having a robot in their personal space. Martinengo et al [41] highlighted empathic responses and integrated CBT exercises as facilitators, while the lack of variety in conversational personas and inability to handle crisis scenarios emerged as significant barriers. Plakun [50]stressed the importance of theory-grounded techniques, such as CBT and relaxation exercises, as facilitators but noted the lack of true empathy and privacy concerns as major hindrances to AI effectiveness.

Russo et al emphasized that digital tools reduced therapy inequalities, appealing to tech-savvy users; however, the small sample sizes and the cohort’s limited understanding of AI’s advancements in therapeutic contexts constrained their ability to fully assess the situation [51]. Prescott and Hanley [52] found that “socially skilled” robots enhanced elder engagement, although speech recognition challenges and cognitive impairments posed obstacles. It was also difficult, according to Goldberg et al [49], to maintain engagement with fully automated operations that lacked human assistance. Dropout rates were occasionally caused by the imagined therapeutic tie being reduced by the lack of a direct human connection, empathy, or prompt feedback [49]. Lastly, Ta-Johnson et al [53] highlighted the chatbot’s role as a “safe space” and its ability to favor long-term interactions, though limited sample sizes and insufficient motivation assessments hindered deeper understanding.

### Quality Assessment of the Identified Studies

Although the studies varied in their design, all were deemed to be of moderate to high quality with minimal risk of bias. However, common challenges included small sample sizes and limited external validity, which were among the most frequently noted issues in the analyzed studies. Quality assessment for each individual study is found in Multimedia Appendix 3.

### Definition of the DTA

Summarizing the abovementioned findings and integrating these concepts, a first definition of the DTA can be established as follows:

The Digital Therapeutic Alliance (DTA) refers to the collaborative relationship and emotional connection between a user and an artificial intelligence (AI)-driven psychotherapeutic tool, encompassing goal alignment, task agreement, therapeutic bond, user engagement, and the facilitators and barriers that influence therapeutic outcomes.

## Discussion

### Principal Results

This integrative review aimed to identify the key concepts in a DTA between AI-driven psychotherapeutic interventions and the user in the context of mental health interventions and provide an initial definition of DTA using these concepts. A total of 23 studies were fully analyzed, and 5 key components such as goal alignment, task agreement, therapeutic bond, user engagement, and the facilitators and barriers that mediate these relationships were observed. The studies were overall of moderate to high quality.

### Comparison With Prior Work

Goal alignment, an essential component of traditional therapy, emerges as equally relevant in digital interventions. Studies highlighted the importance of integrating structured frameworks, such as behavior change theories and SMART goals, to promote shared understanding and collaboration between users and chatbots. These findings are consistent with Horvath and Luborsky [15], who emphasized goal alignment as a predictor of treatment success in traditional therapeutic contexts. Interestingly, digital tools like chatbots simplify goal-setting through automated initiation and measurement, as reported in studies by Aggarwal et al [37] and Beatty et al [25]. However, the findings also reveal challenges in aligning goals with user expectations, which could limit engagement if not adequately addressed, echoing prior concerns about designing interventions in AI-based therapy [8].

The therapeutic bond in the DTA, while conceptually similar to human-therapist relationships, manifests uniquely in digital tools. Emotional connection, empathy, and trust are important factors, as highlighted in studies by Beatty et al [25] and Liu et al [44], which found that conversational agents with empathic tones and anthropomorphic features enhance the bond. However, as noted by Plakun [50], the inability of AI to replicate the emotional depth and transference of human therapists remains a significant barrier. These insights align with literature suggesting that perceived empathy and warmth are central to establish trust and engagement in digital tools, perceived empathy being more important than the real ability of the AI-driven intervention to feel empathy [22]. Although encouraging, issues like rule-based replies and a lack of subtlety in interpreting user emotions highlight the necessity for more technology developments to improve the therapeutic relationship in AI-driven solutions.

User engagement was found to be influenced by both design elements and user perceptions. High initial engagement driven by accessibility and nonjudgmental interfaces, as reported by Alfano et al [54], mirrors earlier findings on the role of usability in DMHIs. However, as Goonesekera and Donkin [57] noted, engagement tends to decline when users do not perceive immediate benefits, highlighting the importance of maintaining long-term motivation. These findings resonate with studies emphasizing the critical role of user engagement in predicting adherence and outcomes in digital interventions [12]. Additionally, themes such as technical reliability, personalization, and responsiveness emerged as key facilitators of sustained engagement, suggesting that addressing these elements could improve adherence in future interventions.

Finally, the review underscores the interplay of facilitators and barriers in shaping the DTA. Facilitators such as anonymity, flexibility, and empathic design were identified across multiple studies, aligning with prior research advocating for user-centered design in DMHIs [11,25]. However, barriers like limited conversational capabilities, privacy concerns, and technical errors, as noted by Chan et al [62] and Russo et al [51], remain persistent challenges. These barriers not only hinder engagement but also undermine user trust. Addressing these barriers requires robust ethical guidelines and technological improvements to address these limitations.

As digital tools evolve, integrating user feedback and advancing AI capabilities will be necessary in overcoming these barriers and optimizing the DTA [63]. However, this evolution raises critical ethical questions, such as “Should therapy be AI-driven?’’ particularly concerning aspects like security, privacy, efficacy, inclusivity, and maintaining a user-centered approach [46]. Vilaza and McCashin [64] have emphasized the importance of implementing evidence-based and empirically tested interventions to ensure that automated therapy genuinely contributes to the improvement of mental health outcomes. Also, earlier findings on DTA support that it may be possible to increase the efficacy of tools like smartphone applications and enhance user adherence by evaluating and improving the DTA [65]. Moreover, applying an ethical framework to the development of these technologies appears essential to ensure accountability and responsibility from AI-developing companies in the field of mental health [66].

### Limitations

It is important to recognize the limitations of this study. First, it is difficult to draw broad conclusions that go beyond the initial definition of a DTA because of the included studies’ variety in terms of design, demographics, and intervention type. Although the analysis made an effort to integrate the findings, differences in the approaches and results could restrict how broadly the findings can be applied. Second, the majority of the research used self-reported metrics to assess ideas like engagement, therapeutic connection, and results, which are prone to response bias and might not fully represent the subtleties of user experiences. It is also important to note that this study included only articles in French or English to limit interpretation bias, which could limit the external validity of the findings in other contexts. Finally, the reviewed literature did not include longitudinal investigations, which limit our understanding of the DTA’s long-term viability and efficacy.

### Conclusions

This integrative review attempted to define the emerging concept of a DTA, offering a preliminary framework for its definition and components. By integrating insights from 28 studies, this work highlights the important role of key elements such as goal alignment, task agreement, therapeutic bond, user engagement, and the interplay of facilitators and barriers. These components mirror the foundations of traditional therapeutic alliances while adapting to the unique dynamics of AI-driven psychotherapeutic tools.

The findings emphasize that, while digital interventions hold promise for enhancing accessibility and engagement in mental health care, challenges such as limited emotional depth, personalization, and ethical concerns persist. Addressing these barriers through technological innovation and user-centered design will be necessary in advancing the DTA. Furthermore, aligning these interventions with evidence-based frameworks, such as CBT or SMART goals, can improve therapeutic outcomes and user satisfaction.

Importantly, the DTA highlights the potential for digital tools to replicate key therapeutic mechanisms, such as empathy, trust, and collaboration, even if in a different context. However, as this field evolves, further research is required to standardize measurement tools, validate the framework across diverse populations, and assess the long-term impact of DTAs on mental health outcomes.

In conclusion, this study provides a foundational step toward conceptualizing the DTA, emphasizing its importance in bridging gaps in mental health care delivery. By addressing its limitations and advancing its understanding, the DTA could play a transformative role in the future of DMHIs.

## Supplementary material

10.2196/69294 Multimedia Appendix 1 Electronic search strategy for the integrative review conducted.

10.2196/69294 Multimedia Appendix 2 PRISMA for Scoping Review checklist.

10.2196/69294 Multimedia Appendix 3 Integrative review study selection detailed results.

## Abbreviations

AI: artificial intelligence
CBT: cognitive-behavioral therapy
DMHIs: digital mental health interventions
DTA: digital therapeutic alliance
PRISMA: Preferred Reporting Items for Systematic Reviews and Meta-Analyses
SMART: Specific, Measurable, Achievable, Relevant, and Time-Bound
WAI-SR: Working Alliance Inventory-Short Revised
