# Supplementary material for: Does the Digital Therapeutic Alliance Exist? Integrative Review
Source: JMIR Ment Health. 2025 Feb 7;12:e69294. doi: 10.2196/69294 (PMC11830484; doi:10.2196/69294)
Supplement: Multimedia Appendix 1 [file mental-v12-e69294-s001.docx]

**Supplementary Online Content**

Malouin-Lachance A, Capolupo J, Laplante C, Hudon A. Does the Digital Therapeutic Alliance Exist?: An Integrative Review

**Multimedia Appendix 1.** Electronic search strategy for the integrative review conducted.

**Multimedia Appendix 1. Electronic search strategy for the scoping review conducted.**

| **Database; Search** | **Search Terms** |
| --- | --- |
|  |  |
| PubMed; k= 902 | (("Artificial Intelligence"[MeSH] OR "Machine Learning"[MeSH] OR "Natural Language Processing"[MeSH] OR "Telemedicine"[MeSH] OR "Mobile Applications"[MeSH] OR "artificial intelligence" OR "AI-powered" OR "machine learning" OR "deep learning" OR "chatbot*" OR "virtual therapist*" OR "AI-driven" OR "AI-powered intervention*" OR "digital mental health intervention" OR "online psychotherapy")) AND ("therapeutic alliance" OR "therapeutic relationship" OR "working alliance" OR "therapist-client relationship" OR "therapist-patient relationship" OR "engagement in therapy" OR "rapport" OR "trust" OR "collaboration" OR "Professional-Patient Relations"[MeSH]) AND ("psychotherapy"[MeSH] OR "psychological therapy" OR "cognitive behavioral therapy" OR "CBT" OR "mental health treatment" OR "digital psychotherapy") AND Humans[MeSH Terms]) |
| Web of Science; k= 106 | TS=(("artificial intelligence" OR "machine learning" OR "chatbot*" OR "AI-driven" OR "online psychotherapy" OR "digital mental health" OR "teletherapy" OR "telemedicine" OR "mobile app*" OR "digital intervention*")  AND  ("therapeutic NEAR/3 alliance" OR "working NEAR/3 alliance" OR "therapeutic NEAR/3 relationship" OR "therapist NEAR/3 client relationship" OR "therapist NEAR/3 patient relationship" OR "engagement NEAR/3 therapy" OR "rapport" OR "trust" OR "collaboration")  AND  ("psychotherapy" OR "mental health treatment" OR "cognitive behavioral therapy" OR "CBT" OR "psychological therapy")) |
| PsycInfo; k = 49 | (TI OR AB OR KW(("artificial intelligence" OR "AI" OR "machine learning" OR "deep learning" OR "chatbot*" OR "virtual therapist*" OR "digital health" OR "e-mental health" OR "online psychotherapy" OR "teletherapy" OR "telehealth" OR "mobile app*" OR "digital intervention*" OR "technology"))) AND (TI OR AB OR KW(("therapeutic alliance" OR "working alliance" OR "therapeutic relationship" OR "therapeutic process" OR "relationship in therapy" OR "therapist-client relationship" OR "therapist-patient relationship" OR "engagement in therapy" OR "client engagement" OR "rapport" OR "trust" OR "collaboration"))) AND (TI OR AB OR KW(("psychotherapy" OR "psychological therapy" OR "mental health treatment" OR "cognitive behavioral therapy" OR "CBT" OR "digital psychotherapy"))) |
| Google Scholar; k= 237 | ("digital therapeutic alliance" OR "online therapeutic relationship" OR "virtual therapeutic alliance")  AND  ("artificial intelligence" OR "AI" OR "machine learning" OR "chatbot" OR "virtual therapist")  AND  ("psychotherapy" OR "cognitive behavioral therapy" OR "CBT")  intitle:("therapeutic alliance" OR "artificial intelligence" OR "digital") |
