# Supplementary material for: Does the Digital Therapeutic Alliance Exist? Integrative Review
Source: JMIR Ment Health. 2025 Feb 7;12:e69294. doi: 10.2196/69294 (PMC11830484; doi:10.2196/69294)
Supplement: Multimedia Appendix 3 [file mental-v12-e69294-s003.docx]

**Supplementary Online Content**

Malouin-Lachance A, Capolupo J, Laplante C, Hudon A. Does the Digital Therapeutic Alliance Exist?: An Integrative Review

**Multimedia Appendix 3.** Integrative review study selection detailed results.

This supplementary material has been provided by the authors to give readers additional information about their work.

**Table S3. Integrative review study selection detailed results.**

| **Authors** | **Sample/Population** | **Type of intervention** | **Agreement on therapeutic goals** | **Agreement on therapeutic tasks** | **Therapeutic Bond** | **Engagement** | **Facilitators** | **Challenges** | **Outcomes** | **Quality Assessment** |
| --- | --- | --- | --- | --- | --- | --- | --- | --- | --- | --- |
| Aggarwal et al., 2023 [37] | Systematic review including adults age 18-60+ with specific condition like obesity, smoking or substance use disorders and studies in the general population. | AI-Powered Chatbots | Integration of behavior change theories, including goal setting, contributed to better primary and secondary outcome. | N/A | N/A | Established mainly by frequence of messages between chatbot and user, but was not consistent between the studies.   "User engagement is dependant on the chatbot's ability to understang user's background, build a relation, be persuasive and offer quick feedback" | Free flow conversations rather than rule-based or constrained conversation with AI chatbots enhanced user experience (need for personalization) | N/A | This review found that AI chatbots were efficacious in promoting healthy lifestyles, including physical exercise and diet (6/15, 40%), smoking cessation (4/15, 27%), treatment or medication adherence (2/15, 13%), and reduction in substance misuse (1/15, 7%).  - Feasibility metrics differed across studies, and there was strong evidence regarding decrease in engagement rates over time - Acceptability : mixed results, the overall satisfaction rate of chatbots was less than 50%.  - Reliability : mixed results, some considered AI efficient and other reported a lack of personal connection with Chatbots | High |
| Alfano et al., 2023 [52] | Focused on population aged 16-25) | Digital Psychotherapy | N/A | N/A | N/A | High initial engagement due to their ease of access and perceived lack of judgment, but dropout rates are a challenge, especially when users don't see quick results. | Being comfortable with digital technology facilitates engagement with AI-powered therapy (concerning adolescents) | Digital therapies face challenges, including machines' inability to understand the emotional, symbolic, relational, and anthropological dimensions of data (C1), limiting their capacity to interpret human experiences fully. Additionally, concerns about data protection (C2) highlight the need for robust privacy measures in these interventions. | N/A | High |
| Anisha et al., 2024 [53] | Scoping review including 43 studies Adults and elderly individuals with noncommunicable diseases (NCDs), especially in higher income countries. | AI-Powered Chatbots | N/A | N/A | N/A | Anthropomorphic CAs are preferred and yielded a higher likelihood of intervention compliance than the machinelike chatbot. | - Strong social presence and emotional closeness are regarded as key facilitators of favorable attitudinal and behavioral outcomes. | Conversational agents (CAs) face significant limitations, including their inability to generate personalized responses (C1) and their lack of natural, empathetic communication due to difficulties in understanding human context and emotions (C2). Additionally, concerns about data privacy (C3) and patient safety, particularly their inability to recognize urgent situations (C4), are frequently overlooked in studies. | N/A | High |
| Beatty et al., 2022 [38] | **Quantitative analysis:** n= 1205 participants that completed the PHQ-4, and were eligible those who responded ≥3 for the first two questions (anxiety) or ≥3 for the second two questions (depression).   n=226 for the second administration of the test 3 days later  **Qualitative analysis :** n =950 based on users conversations using relevant keywords. | Digital Psychotherapy | Measured by WAI-SR subscale goals. Users and Wysa collaborate to set therapeutic goals related to mental health improvement | Measured by WAI-SR subscale tasks | Measured by WAI-SR subscale bond. Users expressed strong emotional connections in the first assessment comparable to traditional in-person CBT. | 73.8% of participants continued using Wysa after the first assesment (average 6 conversations over 14 days).   Engagement improved the longer the users interacted with Wysa, withe the bond subscore increasing by the second assessment. | Anonymous  flexibility and autonomy provided by free text CA fostering the user's autonomy | Limitations in understanding lead to some frustration Perceived ineffectiveness | WAI-SR - each item is rated on a 5-point scale rating from 1 (strongly disagree) to 5 (strongly agree)  **First assessment :  *Bond subscore: Mean = 4.05 (SD 0.91)*** Goal subscore: Mean = 3.7 (SD 0.86) Task subscore: Mean = 3.5 (SD 0.77) Total score: Mean = 3.75 (SD 0.80)  **Second assessment :  *Bond subscore: Mean = 3.98 (SD 0.94)*** Goal subscore: Mean = 3.62 (SD 0.93) Task subscore: Mean = 3.33 (SD 0.84) Total score: Mean = 3.64 (SD 0.81)  Bond subscale comparable to face-to-face study (mean around 4.0)  Wilcoxon Signed-Rank test show no statistically significant change in participants' perception of the therapeutic alliance between the first and the second assignement | High |
| Chan et al., 2022 [58] | 2409 users recruited from multiple sources at risk of ED on the Weight Concerns Scale. Some users were anonymous. | AI-Powered Chatbots | N/A | N/A | N/A | N/A | N/A | Inappropriate Reinforcement: Chatbot responses sometimes reinforced harmful behaviors or provided overly generic positive feedback. Failure to Address Questions: Instances of ignoring or being unable to answer users' questions effectively. Lack of Clarity: Responses were occasionally unclear, missing instructions, or failing to provide necessary clarifications. Contextual Misunderstandings: Difficulty in understanding context, such as misinterpreting quotes containing swear words or failing to address inappropriate content. Technical Errors: Occasional technical glitches affecting functionality. | N/A | High |
| Easton et al., 2019 [59] | 6 adults with a COPD diagnosis and 5 health professionnals, and 12 additionnal adults with COPD for the acceptability and engagement assessment | Personalized Therapy | N/A | N/A | N/A | N/A | F1. To be recognizably human in appearance and to be able to personalize Ava to some dregree F2. Have option for voice or text only. F3. Desire for a conversational discourse but also education provision. | N/A | Overall rating of the system 73.75 50% of the participants (4/8) strongly agreed with the statement "i think i would like to use this system frequently"  Suggesting a potential for a relationship of sorts between human and autonomaux agents. | Moderate |
| Entenberg et al., 2023 [41] | 170 parents from Argentina, mainly female (95,3%), married (70%), university or tertiary level of education (75,9%) and employed (81,7%).  Experimental group 89 parents Control group 81 parents | AI-Powered Chatbots | Established by the Chatbot at the beginning of the conversation | N/A | N/A | Established by the number of messages and characters sent | Brief conversation well suited for parents | N/A | Completion rate lower (66.3%) than the one reported in human supported digital parenting intervention and a focused 2h face-to-face group discussion.  High satisfaction and acceptability. | High |
| Escobar Viera et al., 2023 [54] | 20 ruralliving LGBTQ+ youth aged 14-19 who screened positive (16/20) for social isolation. | Digital Psychotherapy | N/A | N/A | N/A | established by the number of messages sent:  Participants sent an average of 49,3 messages to the chatbot | chatbot asked for name (trans were not called by their dead name), quick to respond, pleasant design for teens | The Chatbot was mostly educationnal on social media interactions The content was limited Limited ability to engage in conversations | Non-significant changes in scores of - perceived isolation - depressive symptoms - social media self efficacy | High |
| Forman-Hoffman et al., 2023 [43] | 255 participants (11% resided in a nonmetropolitan or rural area "MUA") (40% lived in a medically underserved area and 55% in a mental health provider shortage area "MHPSA") | Exploratory or Review Studies | Evaluated via the therapeutic alliance score. | Evaluated via the therapeutic alliance score. | Evaluated via the therapeutic alliance score. | Utilization (opened app on at least half of the weeks 4/8): - 76% MUA no - 72% MUA yes - 71% MHPSA no - 77% MPHPSA yes | Smartphone-based app ("previous research has found Woebot-based DMHIs to be feasible, acceptable and efficacious among various populations") | N/A | Participants living in a MUA had several higher therapeutic alliance scores | High |
| Goldberg et al., 2020 [44] | Recordings from 1,235 sessions of 386 clients seen by 40 therapists at a university counseling center were processed using automatic speech recognition software. Machine learning algorithms learned associations between client ratings of therapeutic alliance exclusively from session linguistic content. | General AI Healthcare Tools | Done between humans. | Done between humans. | Done between humans. | N/A | N/A | Therapeutic Alliance has more depth than only linguistic content, it can make it hard for machine learning to predict  Modest sample size  Rating of assessment of alliance were made retrospectively | "The predictions of three out of the four models are significantly better than chance (Spearmańs ρ > .00, p < .01)."  ML could potentially tell a therapist when alliance seems unsatisfactory to the patient via the linguistic content. Sometimes patients are unsatisfied but won't say so due to social desirability or else. | High |
| Goonesekera et al., 2022 [55] | 29 users who completeted a 14-day program run by Otis | Digital Psychotherapy | N/A | N/A | N/A | "expectancy, participants reported that, although the “check-ins” were short, conversing with the chatbot daily was tedious and the expectation of participant time was too much. Social engagements, fatigue, and daily tasks were commonly cited as distractions."  The final sample completed 70% of the intervention. | - Interactivity: anthropomorphism - Appearance - Perceived benefits - Content delivery | - Interactivity: high performance activity - Effort expectancy - technical difficulties - Decision tree-based chatbot: limited discussions | There was no significant reduction in health anxiety. However, the intervention was associated with improvements in: Decreased generalized anxiety, Enhanced management of generalized anxiety, Personal well-being, and Quality of life. | High |
| He et al., 2022 [39] | 153 smokers randomly assigned to MI-style chatbot ir nuetral chatbot | AI-Powered Chatbots | Measured using the *Working Alliance Inventory-Short Revised* | Measured using the *Working Alliance Inventory-Short Revised* | Measured using the *Working Alliance Inventory-Short Revised* | Via the User Engagement Scale (O’Brien HL, Cairns P, Hall M. A practical approach to measuring user engagement with the refned user engagement scale (UES) and new UES short form. Int J Hum Comput Stud. 2018;112:28–39.)  More engagement with motivaltional-interviewing chatbot | Low dropout rates | - Validity os the self-reported motivation to quit smoking - the chatbox did mistakes during the experiment (eg. replying with a wrong answer) -developping a relational feelings such as engagenebt nught need a linger time than 2 sessions | Interacting with a chatbot about smoking cessation can motivate smokers to quit The therapeutic alliance between participant and chatbox increased after two sessions Perceived communication competence was positively correlated with engagement at both T1 r (151)=0.47, p<0.001, and T2 r (151)=0.63, p<0.001. Last, for perceived empathy, no main efect of time was found F (1,151)=0.79, p=0.377, nor an interaction between condition and time F (1,151)=0.08, p=0.779. | High |
| Hocking et al., 2024 [45] | Adults with TBI receiving brain injury rehabilitation and clinicians providing this care. | AI-Powered Chatbots | Use of the SMART goals framework. | Use of the SMART goals framework. | N/A | The two patients had good and stable engagment | - Straighfoward, not a lot of effort needed from the patient  - provides check-ins to see if patients on target with their goals - motivating | - client forgets what goals they setted - no subjective goals (eg. confidence to talk to people) | Usability and Acceptance:  RehabChat demonstrated good usability with a mean SUS score of 76.25 (range: 67.5–82.5), indicating positive user acceptance. Impact on Well-being:  One patient showed improvement in well-being, while the other patient's well-being remained stable. | High |
| Inkster et al., 2023 [56] | Real Wysa users who reported going through a maternal event during their conversation with the app (grouped in 4 maternal events: pre-pregnancy, pregnancy, perinatal, postpartum) | AI-Powered Chatbots | N/A | N/A | N/A | Based on their number of active session-days with the CA between two screenings | N/A | N/A | Effectiveness (n=51, quantitative):  Users with high engagement showed a significant reduction in depressive symptoms compared to those with lower engagement (p = .004). Engagement (n=10, qualitative):  97% of users reported high or moderate satisfaction, indicating positive user engagement and receptivity to the intervention. | High |
| Jeong et al., 2023 [40] | 70 participants (- 10 who withdrew)  (1- live in the US 2- internet acces 3- no auditory/visual/oral disability 4- were not receiving treatment for depression) | AI-Powered Chatbots | Measured using the *Working Alliance Inventory-Short Revised* | Measured using the *Working Alliance Inventory-Short Revised* | Measured using the *Working Alliance Inventory-Short Revised* |  | Patients found the robots engaging and appreciated their positive reinforcement, enjoying both the wellness activities and the intervention materials, including the Positive Psychology Interventions (PPI) content. | Patients needed time to adapt to having robots in their homes, and the study's findings were influenced by external factors, making it difficult to isolate the robot's specific effects. Additionally, reliance on self-reported measures and interviews introduced potential response bias. | Intervention Impacts:  Both coach-like and companion-like robots had positive effects on intervention outcomes. The companion-like robot yielded the largest improvements in psychological well-being compared to the coach-like robot. Working Alliance:  The control condition showed a significantly lower working alliance compared to the experimental conditions: Control: MD=1.958 Coach-like: MD=3.333 Companion-like: MD=3.750 χ²(2)=14.409, p<0.001. Coach-like Robot Effects:  Significant improvements were observed in: Overall well-being (RPWS_total: coef=0.023, p=0.048) Overall affect (PANAS_total: coef=0.074, p=0.004) Significant reduction in negative affect (PANAS_NA: coef=−0.050, p=0.004). Companion-like Robot Effects:  Significant improvements were observed in: Overall well-being (RPWS_total: coef=0.033, p=0.030) Personal growth (RPWS_PG: coef=0.038, p=0.036) Self-acceptance (RPWS_SA: coef=0.047, p=0.014) Confidence for change (RR_change: coef=0.105, p=0.010) Significant reduction in negative affect (PANAS_NA: coef=−0.036, p=0.004). Human-Robot Rapport:  Participants' rapport with the robot was positively associated with improvements in psychological well-being. This finding suggests that the human-robot rapport may play a role similar to the clinician-patient rapport, which is known to predict treatment outcomes in traditional therapeutic settings. | High |
| Kettle et al., 2023 [57] | Study 1: 1468 unique Reddit posts on user experience of mental health apps with CA functionnality (Unstructured collection of people opinions and unrestricted sample size)  Study 2: 19 participants (11 women, 8 men, mean age= 19,5 years) recruited through the George Mason University Psychology department's student research portal, SONA, or through email recruitment and word to mouth (criterias: 18-24 yeard of age, have a working mobile phone, normal vision + hearing abilities, able to download an application to their phone) | AI-Powered Chatbots | N/A | N/A | N/A | Study 1:  *7. Agent connexion*: connecting with the agent lead to greater app usage  8: *initial usage motivation*: curiosity, loneliness, mental health support and previous AI experience 9. *Technical features*: Other enjoyed the accessibility to interact with the chatbot on multiple devices  Study 2:  More specific questions kept focused engagement to the chatbot. | Study 1:  Chatbot personnality seems to play a large role in app engagement  Study 2: by having participants interact with two chatbots, the research gained insight into themes that reflected chatbot distinctions wich would not otherwise have been identified if only engaging with one control condition | Study 1: The price of subscription costs expensive outside of the USA (developpers should set different price expectations for international users) | Study 1:  **11 emerging themes:** 1. *accessibility:* mixed feelings about premium versions, but favorable regarding freemium VS human-rovided therapy 2. *Communication/conversation style:* mixed preferences between individuals 3. *AI experience:* specific experiences influenced their continued use 4. *Anthropomorphism:* many enjoyed anthropomorphic features (some perceived the chatbot's avatar as a child and found venting to this perception problematic) 5. *Acceptance/Understanding:* people understood the AI was not human, some questionned how the AI determined what was considered healthy 6: *App function:* 7. *Agent connexion:* connecting with the agent lead to greater app usage 8: *initial usage motivation:* curiosity, loneliness, mental health support and previous AI experience 9. *Technical features:* technical difficulties leading to negative experiences. Other enjoyed the accessibility to interact with the chatbot on multiple devices 10. *Miscellanous:* specific comments included lack of confidence in the developpers addressing the user wants, 11. *Privacy and rights:* users discountinued the use of the application because of concerns regarding nvasion of privacy, the safety of the application and the privacy of users' data  Study 2:  User response: when comparing the 2 modalities, participants noted their preference toward a mixture of being able to freely respond while also being offered guided responses  Question specificity: asking more specific questions afforded more reflection, accountability and kept focused engagement to the chatbot  CA reponse time and lenght: mixture of preferences (immediate VS delayed, single large response VS smaller)  Conversation flow control; enjoyed the possibility to focus on one health domain, but some would like to control the conversational pathway in initial uses  Perceived CA Role: chatbot felt like a friend for some or more practionner-like for others  Digital Images and imperfect punctuation: positive to the use of emojis, choice of word and imperfect punctuation made the chatbot feel more human-like  Repetition: Simplifying and repeating participants answer was received favorably  Task Immediacy: confusion when the CA required them to engage in a task immediatly (they may not be in the right headspace)  Calibrated Expectations: early expectation calibration led to more positive experiences  Bidirectionnal Communication only 2 participants (10.5%) commented that interactions were more one-sided (making the chatbot less relatable) | High |
| Liu et al., 2022 [46] | 83 university student from China, 46 of which were female. Participants were between 19 and 28yo (mean age of 23.08) with a PHQ-9 score equal to or greater than 9 | AI-Powered Chatbots | Done between humans. | Done between humans. | Therapeutic alliance mesure with the WAI-SR score is shown to be higher with the conversational AI than with the bibliotherapy. | Similar between the chatbot use and the bibliotherapy | -smartphone based chatbot and easy to assess | Chat-bot was limited since all content needed to be aproved by professionals which created repetitive conversations. - No DST or DPO which limited the personalisation of the therapy -therapy focused on depression only -Lack of participant variety | Effectiveness:  Depression (PHQ-9): The chatbot intervention significantly reduced depression over 16 weeks with a large effect size (d = 0.83, F = 22.89, P < 0.01). Anxiety (GAD-7): Anxiety reduction was significant during the first 4 weeks with a small effect size (d = 0.30, F = 5.37, P = 0.02). Effects on both depression (P < 0.01) and anxiety (P = 0.046) remained significant after Bonferroni correction. Therapeutic Alliance:  The chatbot group demonstrated a significantly stronger therapeutic alliance (WAI-SR) compared to bibliotherapy (t = 7.29, P < 0.01, d = 1.85). No significant difference in client satisfaction (CSQ-8) between the groups (t = 0.88, P = 0.38). Adherence:  Adherence rates were comparable between the groups (chatbot: 1.96 ± 0.70; bibliotherapy: 1.97 ± 0.62; t = 0.68, P = 0.50). The chatbot group experienced a decline in adherence over time, whereas adherence in the bibliotherapy group slightly increased during the first 8 weeks. | High |
| MacNeill et al., 2024 [47] | 68 individuals suffering from diabetes or chronic arthritis | Behavioral Change Tools | N/A | N/A | Present for some, they felt like it was understanding, reassuring and non judgemental but others felt like the conversationnal issues kept them from developing a bond | N/A | -Constant availability -Chatbots were perceived as understanding, non-judgmental, and described as kind and reassuring. -Automatic check-in messages were appreciated. -Therapeutic exercises were considered useful and quick to complete. | -Conversations felt unnatural, general, and repetitive. -The bot was unable to understand the nuances between mental health issues and physical suffering. -Design difficulties and technical problems made the bot unpleasant for some users. -Short study duration (4 weeks). | The treatment group showed a significant decrease in depression and anxiety by week 2, continuing through week 4, while the control group saw no meaningful changes. There was no significant effect on stress in either group. | High |
| Martinengo et al., 2022 [42] | 9 conversational agents available to download and aimed at mental health support | AI-Powered Chatbots | Done with the conversational agent. | Done with the conversational agent. | The conversational nature and friendly, empathic tone facilitated engagement. However, rule-based agents limited the bond and tended to create frustration. | Rule-based Conversational Agents (CAs) often provided inadequate responses and repeated requests, making engagement more challenging. | -Empathic responses and integrated CBT exercises -Engaging for individuals who might not have sought medical help but felt depressed -Ability to guide users when a crisis is identified | -The use of only two standardized personas limited the variety of conversations studied. -The bond and other factors were evaluated subjectively by the research team, and the sample size was small. -Suicide risk and crisis were difficult for the Conversational Agents (CAs) to detect and needed to be explicitly stated in the prompts. | Strengths:  AI-powered agents excel in long-term bonding and delivering personalized responses. Lower attrition rates were observed with Conversational Agents (CAs) compared to other mental health apps. Effective in empathic conversations and depression management, particularly for individuals hesitant to seek traditional healthcare. Limitations:  Risk of bias and sometimes offering unverified answers. Inadequate for assessing and managing suicide risk. Conversations can feel incomplete and fail to fully emulate human-patient interactions. | Moderate |
| Plakun, 2023 [48] | None - a psychyatrist and "senior psychodynamic psychotherapist's" opinion | Digital Psychotherapy | N/A | N/A | - The therapeutic bond, along with the transference and countertransference found in human relationships, strongly limits AI capabilities. The problem lies in AI's incapacity to develop true countertransference due to its lack of an individual history intersecting with the patient's.  -Patients may still feel that a relationship is building, given the human-like responses generated by AI. -The lack of authenticity and genuine empathy limits the psychotherapy that can be achieved, resulting in a truly limited therapeutic alliance as we know it. -The placebo effect may be a double-edged sword; while it can be beneficial for some populations, the digital placebo might negatively impact others struggling with engagement issues and the need for authentic human interaction. | N/A | Techniques grounded in theory are essential, including ERP, DBT, relaxation techniques, and CBT. AI is very promising in this aspect. | -The lack of privacy and strict rules regarding AI data makes it difficult for patients to engage in a truly safe space. This limitation is significant, but it may improve with future legislation and an increase in patients' digital knowledge.  -The lack of true empathy and the inability to reflect using past personal life experiences further hinder the effectiveness of AI in therapeutic settings. | -AI therapy is likely to underperform, particularly in forms of therapy that rely heavily on authentic human relationships, such as psychodynamic therapy.  -AI requires strong legislation and practice codes to ensure ethical use. -The challenges may be insurmountable due to the inherent impossibility of establishing a true relationship based on the artificial persona adopted by AI, as well as its lack of genuine emotions, empathy, and the capacity to reflect on real personal life history. | High |
| Prescott et al.,2023 [49] | 35 therapists, 26 of which were trainees. They are 69% female, aged 22 to 39 yo and mostly from UK. Only 1 participant had previously worked with AI therapy. | General AI Healthcare Tools | N/A | N/A | The therapeutic bond was rated as the most challenging aspect of AI use in therapy from the therapists' perspective. They find it hard to believe that a machine could develop the same emotional depth and nuances as a human. | Can facilitate therapy by reaching hard-to-access individuals and contribute to a slight reduction in inequalities and stigma. | Patients have historically been more inclined to appreciate technology and AI chatbots than therapists. | -Small sample size -Uk sample only -Limited understanding of the progress and possibilities of AI within therapeutic settings | -Therapists are cautious and deeply question the ability of AI to form a therapeutic bond. -General knowledge of AI in therapeutic settings is limited. -Despite being very critical, therapists seem open to what AI and machines can bring to the future of therapy. | Moderate |
| Russo et al., 2019 [50] | 12 selected published artices between 2000 and 2017. Inclusion criterias were : 1) Use of conversational agents, dialogue systems or language processing tools for  people with cognitive impairments 2) Age ≥ 60 years 3) Diagnosis of dementia according to NIAAA and DMS-5 4) Presence of tests or experiments with qualitative or quantitative results. | Exploratory or Review Studies | N/A | N/A | Although not directly evaluated, one of the included articles underscored elders' propensity to be more expressive when interacting with more sociable robots compared to task-oriented, less socially communicative robots. | x | -A slow speech rate, closed-ended questions, repetitions, verification questions, and reduced syntactic complexity aid in elders' understanding.  -"Socially skilled" robots offer better engagement and positively impact user acceptance. | -Limited accuracy of speech recognizers. -Speech abnormalities and disfluencies in older adults with dementia pose challenges in speech recognition and language processing. -Algorithms and AI bots need to be adapted to account for the understanding limitations of dementia patients. | 1) More socially communicative abilities made patients feel more comfortable and expressive. 2) Patients are less verbal with machines than with human therapists when asked the same questions, but interactions with machines could still be valuable when no human is present. 3) Better results are observed when limited information is given to impaired elders, with the bot acting as a virtual agent. 4) Perception, interpretation, and conveying emotions through vocal effects are essential. 5) Assessing the cognitive status of people with dementia can be done by identifying metrics and using speech processing and NLP bots to discriminate between patients with and without cognitive impairment. 6) Speech remains the most powerful and effective communication method. Bots that can utilize NLP to understand a patient's emotional state through voice and speech characteristics will aid in social assistance and cognitive impairment diagnostics. | High |
| Ta-Johnson et al., 2022 [51] | 66 "Replika" chat bot users, 54% men, mostly single american and white from 17 to 68 years old. | AI-Powered Chatbots | N/A | N/A | Although not directly discussed, users seem to build confidence in the chatbot and use it to discuss things they feel too scared to tell others. Users seek its (Replika's) help for mental health issues, but also as someone to exchange thoughts about their lives and feelings. Based on the responses, a form of bond is present, resembling a therapeutic relationship concerning mental health issues. | Users seem to engage frequently and steadily on various topics. They feel compelled to return to the chatbot and provide it with updates about their lives and emotions. | -The chatbot is qualified as a "safe space." -It is easy to use, and a sort of "friendship" develops due to Replika's ability to interact with users on a wide variety of subjects. | -The study aimed to explore the positives of human-chatbot interactions and the motivations behind them. -It does not assess why users continue to use Replika if those reasons differ from their initial motivations. -The sample size is small. | -A chatbot capable of offering mental and physical health support is more efficient and better meets people's needs, as it helps foster a long-term relationship with the AI. -The usage of social chatbots shapes how people perceive chatbots and AI. Interest in how they work and what they can do is a significant motivation and could influence the social narrative surrounding AI. -Users appreciate being able to discuss various subjects, including mundane topics, but especially emotional and complex ones that they feel afraid to communicate with other humans. -Chatbots are used as a means of validation and to reduce loneliness, which could potentially help prevent some mental health issues. | Moderate |
| Macrynikola et al., 2024 [60] | 82 adults (73% White, 64% cisgender women, mean age 41) | Digital psychotherapy | Measured using WAI-SR and DWAI scales | Linked to engagement in cognitive-behavioral strategies | Both therapeutic and digital alliance were predictive of outcomes | Increased ER self-efficacy and reduction in symptoms | Digital tools, validated scales, hybrid model | Limited understanding of digital mechanisms | Reduction in depression and anxiety symptoms | High |
| Brotherdale et al., 2024 [61] | 17 individuals utilizing mental health apps | Exploratory or Review Studies | Explored in interviews, lower expectations compared to in-person | Choice and empowerment were key themes | Connection with 'another' was reported | Accessible and safe; loyalty and trust facilitated usage | Human voice, validating statements, personalized interfaces | High attrition rates, lack of personalization | Conceptualization of digital therapeutic alliance | Moderate |
| Golderberg et al., 2022 [62] | 290 meditation app users and 314 trial participants | Behavioral Change Tools | Assessed via DWAI, aligned with digital context | Aligned with perceived app effectiveness and task adherence | Evaluated in the DWAI, emotional bond present but weaker digitally | DWAI predicted higher app utilization and engagement | Perceived app effectiveness, emotional connection | Measurement challenges, anthropomorphic item critiques | Validation of DWAI, predictive validity for psychological distress | High |
| Doukani et al., 2024 [63] | 945 participants (mean age 38.96, 68.2% female) | Digital Psychotherapy | Significant association in blended CBT, higher scores than TAU | Higher agreement observed in bCBT group | Significant bond scores in bCBT; usability enhanced bond | Good system usability enhanced engagement | Blended delivery, usability heuristics | Variability in alliance ratings across settings | Higher WAI scores in bCBT linked to improved PHQ-9 scores | High |
| Ashur et al., 2024 [64] | Parents from 68 families in a digital parent training program | Personalized Therapy | Addressed by enhanced program features in digital intervention | Enhanced quality features supported task-related alignment | Sense of relatedness unique to digital interventions | Higher engagement in enhanced quality programs | Therapeutic persuasiveness, quality enhancement | Inconsistencies in traditional vs digital TA associations | Improved child behavior with enhanced program | High |

AI: Artificial Intelligence; bCBT: Blended Cognitive Behavioral Therapy; CBT: Cognitive Behavioral Therapy; CA: Conversational Agent; DTA: Digital Therapeutic Alliance; DWAI: Digital Working Alliance Inventory; ER: Emotion Regulation; ETAI: eHealth Therapeutic Alliance Inventory; GAD: Generalized Anxiety Disorder; MUA: Medically Underserved Area; NLP: Natural Language Processing; PHQ: Patient Health Questionnaire; RCT: Randomized Controlled Trial; SUS: System Usability Scale; TA: Therapeutic Alliance; WAI-SR: Working Alliance Inventory-Short Revised.
